# A Rare Case of Brucellosis with Multivalvular Endocarditis and Complete Heart Block

**DOI:** 10.2174/011573403X290326240703100925

**Published:** 2024-07-03

**Authors:** Sunil Kumar Gothwal, Kanika Goyal, Ajay Shankar Garg, Bharat Kumar Sahu, Mohit Agrawal, Anurag Mishra, Yogendra Singh, Vetriselvan Subramaniyan, Neelam Singla, Md Sadique Hussain, Gaurav Gupta

**Affiliations:** 1 Department of Medicine, Jawaharlal Nehru Medical College, Ajmer, Rajasthan, India;; 2 Department of Pharmacology, School of Medical & Allied Sciences, G.D. Goenka University, Gurugram, India;; 3 NIMS Institute of Pharmacy, NIMS University, Jaipur, India;; 4 Department of Pharmacology, Maharishi Arvind College of Pharmacy, Ambabari Circle, Ambabari, Jaipur, 302023, India;; 5 Department of Pharmacology, Jeffrey Cheah School of Medicine & Health Sciences, Monash University, Petaling Jaya, Malaysia;; 6 School of Pharmacy, Suresh Gyan Vihar University, Jagatpura, Mahal Road, Jaipur, Rajasthan, 302017, India;; 7 School of Pharmaceutical Sciences, Jaipur National University, Jaipur, Rajasthan 302017, India

**Keywords:** Brucellosis, endocarditis, transthoracic echocardiography, pan systolic murmur, Av block, contaminated aerosols

## Abstract

**Background:**

Brucellosis is a public health concern that affects multiple organs. However, cardiovascular problems arise infrequently, affecting fewer than 2% of cases, typically presenting as endocarditis.

**Case Presentation:**

A 50-year male was admitted with low-grade fever, night sweats, weight loss (13 kg), malaise, and generalized weakness for the past 6 months. On clinical examination, he was febrile with 39.0°C, an average heart rate of 54 bpm, and 100/40 mmHg blood pressure. On cardiovascular examination, S1 and S2 were soft with pan systolic murmur present in the mitral area, and the early diastolic murmur was present in the left third intercostal space. Electrocardiography was suggestive of third-degree heart block with AV dissociation. Transthoracic echocardiography showed mobile vegetations attached to multiple valves- an aortic valve (18.2x11.9 mm) and a mitral valve (2.9x7.5 mm) with perivalvular abscess. He was given oral doxycycline (100 mg B.D.) and rifampicin (600 mg/day); the patient responded, but the AV block did not resolve.

**Conclusion:**

This report has drawn attention to multivalvular involvement and cardiac rhythm abnormalities in Brucellosis (in this case, A.V. dissociation was present) because early diagnosis and treatment can cause a significant decrease in morbidity as well as mortality by appropriate treatment.

## 1 INTRODUCTION

Brucellosis is a bacterial infection caused by intracellular non-motile gram-negative *coccobacilli Brucella*. The most common species affecting humans are *B. abortus* and *B. melitensis*, which are major public health issues [1, 2]. Therefore, patients with chronic fever history of contact with animals and their products should be suspected as differential diagnoses. This is due to non-pasteurized milk consumption, food products, direct contact and treatment of infected animals, conjunctival inoculation, voluntary inoculation, skin cutting, and inhalation of contaminated aerosols [3, 4]. Human-to-human transmission is very rare. It may influence many human organs and body systems. Gastrointestinal, musculoskeletal, genitourinary, respiratory, and hematological processes are the most often affected. Brucellosis is variable in the clinical presentations of mild, moderate, and severe diseases. The average incubation period is 1–4 weeks.

Brucellosis cardiac complications are rare, are in less than 2% of patients, and are typically the most common cause of mortality, as endocarditis [5]. It generally incorporates the aortic valve and allows an immediate valve replacement intervention [6]. Isolated pericardial and myocardial behaviour is not normal and is rarely recorded in the absence of endocarditis [7]. The mechanism of cardiac injury is not well known but may be due to direct invasion or complex immune damage caused by micro-organisms (confirmed by pericardian fluid isolation) [8].

## 2 CASE REPORT

A 50-year-old male, goatherd by occupation, was admitted to the medical department with complaints of low-grade fever with night sweats, significant weight loss of around 13 kg, malaise, and generalized weakness for the past 6 months. He had refuted any unprotected sexual contact or prior tuberculosis infection. There was no history of any drug abuse. However, he had a history of drinking goat's milk, which he had raised. The patient was treated with analgesics and empiric oral antibiotics in regional healthcare facilities and then referred for further examination because of recurrent symptoms.

On clinical assessment, the patient's body temperature was 39.0°C, average pulse intensity 54 bpm, which was regular (on admission), blood pressure was 100/40 mmHg in the right arm, and BMI 18.5kg/m^2^. In physical examination, pallor and clubbing were present, and JVP was normal. The respiratory system was unremarkable (respiratory rate 16/min and SPO2 98% on room air, and lungs were clear on auscultation). At the same time, an abdominal examination revealed mild tenderness in the left hypochondrium with mild splenomegaly. On cardiovascular assessment, S1 and S2 were soft. A high-pitched, soft-blowing pan systolic murmur of grade 3 was present in a mitral area radiating to the axilla, best heard with the diaphragm of the stethoscope in the left lateral decubitus position at the end of expiration. Also, a pan systolic high-pitched soft-blowing grade 3 murmur was present along the left lower sternal border (tricuspid area), which increased inspiration. A high-pitched soft-blowing decrescendo early diastolic murmur was best heard in the 3^rd^ left intercostal space (neo-aortic area) inpatient sitting and leaning forward with breath held in forced expiration. There was no cyanosis, oedema, or peripheral lymphadenopathy, and the rest of the examination was unremarkable. There was no history of hypertension, diabetes, stroke, bleeding disorders, and autoimmune disease. There was no family history suggestive of a similar illness.

### 2.1 Laboratory Investigations

**Fig. (1):**
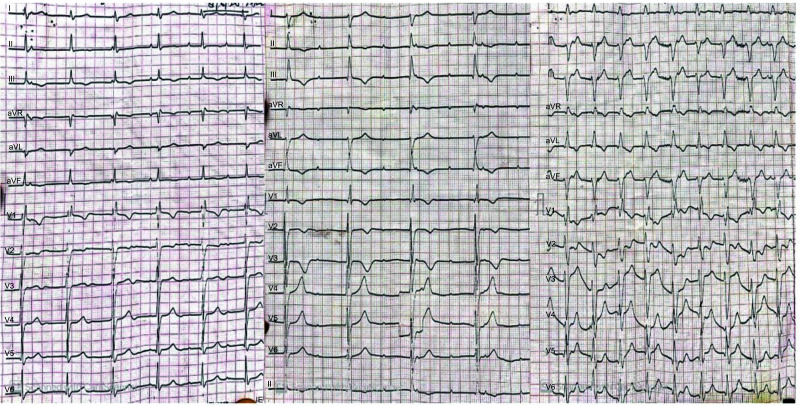
ECGs shows complete heart block with AV dissociation.

**Fig. (2):**
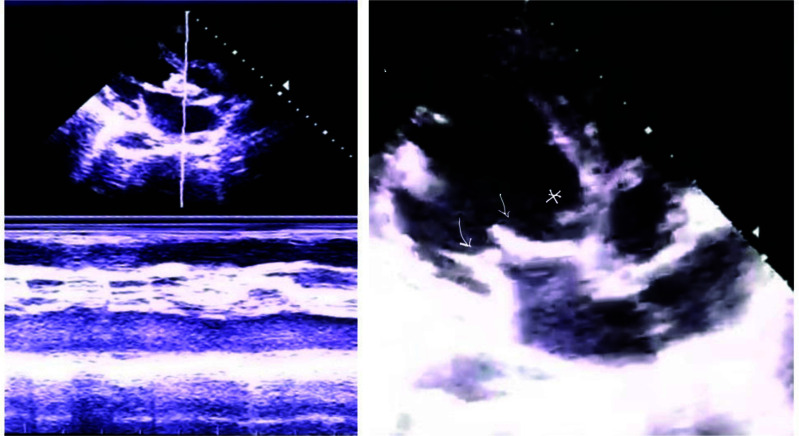
TTE- Long axis parasternal view- in left image M mode shows dense and shaggy echoes on aortic valve (Ao) suggestive of endocarditis with thickened aortic valve and right image shows aortic(asterix) and mitral (arrow) vegetations.

Blood tests showed Hb 8.4 gm/dl, TLC 12700/mm3 and DLC- Neutrophils 82.3%, Lymphocytes 15.4%, platelets 213× 103/mm3, and ESR 50 mm/hr. Liver and renal function assays were normal. Blood biochemistry showed normal fasting sugar (104 mg/dl), calcium (7.7 mg/dl), albumin (2.2 mg/dl), corrected calcium for albumin (9.14 mg/dl), sodium (134 mg/dl), and potassium (4.0 mg/dl). Urine microscopy reported hematuria, 8-10 RBCs/HPF. Chest radiograph was normal, and abdominal sonography revealed splenomegaly with multiple small splenic abscesses of variable size. Blood and urine cultures taken on admission were negative. Electrocardiography (E.C.G.) showed complete heart block and A-V dissociation (Fig. **1**). Transthoracic Echocardiography (T.T.E.) showed mobile vegetations attached to multiple valves-aortic (of size18.2x11.9 mm) and mitral valve (of size12.9x7.5 mm) with perivalvular abscess (Fig. **2**). There was moderate mitral regurgitation with moderate tricuspid regurgitation, severe eccentric aortic regurgitation, and moderate aortic stenosis. The right and left atriums were dilated, and pulmonary artery hypertension was present with normal Left Ventricular (LV) diastolic and systolic ejection fractions.

The theory of Brucellosis has been raised, taking account of the signs and epidemiology of cattle exposure, and brucella Serum Agglutination Titre (S.A.T.) was sent. A brucellosis agglutination test with a 1/320 titer for both B. abortus and B. melitensis was positive; thereby, a Brucella-related endocarditis diagnosis was made. Treatment included beginning with oral doxycycline (100 mg B.D.) and rifampicin (600 mg/day); the patient responded well and become afebrile, and there was an improvement in the appetite, but the A.V. block did not resolve. The patient was referred to the cardiothoracic surgery department for further necessary surgical interventions for A-V block and perivalvular abscess.

## 3 DISCUSSION

Endocarditis is the most common cardiovascular manifestation and most common complication of Brucellosis, leading to mortality. It can seldom involve the pericardium and myocardium and usually affects the aortic valve, while the mitral valve may be involved less frequently and require urgent surgical valve replacement [9-12]. In our case, vegetation was found on both mitral and aortic, along with perivalvular abscess and pulmonary hypertension.

To reach a diagnosis, several medical records, clinical evaluation, biochemical and hematological testing, imaging scans, microbiological assays, and brucella-specific molecular and serological tests are required. In this case, the diagnosis was suspected based on history and positive Brucella serology. A more practical and widely used test for diagnosis is the SAT because of its widespread availability and low cost. SAT detects brucella antibodies to multiple species – B. abortus, B. melitensis, and B. suis. These tests may show false-positive results but support our diagnosis. If treatment starts in the early phase of Brucellosis, the patient may respond well and decrease significant mortality. We used I.M. streptomycin (0.75–1 g daily for 14–21 days), Rifampicin 600 mg/d, and doxycycline (100 mg twice daily for 6 weeks in this case). The patient responded well in terms of no fever, feeling of well-being, and increased appetite. Therefore, WHO recommended an alternative regimen of rifampin (600–900 mg/d) plus doxycycline (100 mg twice daily) for 6 weeks. Brucella endocarditis is usually treated with at least three drugs (an aminoglycoside, a tetracycline, and rifampin) for 4 to 6 weeks. Many experts add ceftriaxone/or fluoroquinolone to reduce the need for valve replacement. However, most cases require valve replacement, as in this case [13].

Pulsed Wave Tissue Doppler Imaging (PW-TDI) has been investigated as a possible method for evaluating the risk of embolism associated with mobile intra-cardiac masses [14]. Traditionally utilized for assessing cardiac function and diagnosing various heart conditions, the use of TDI to predict embolic risk from mobile intra-cardiac masses represents an emerging area of study [15, 16]. In addition to conventional transthoracic echocardiography, PW-TDI can provide valuable incremental prognostic information in cases of infective endocarditis with mobile vegetations. The TDI-derived mass peak antegrade velocity, measured at the level of the mobile portion of the vegetations, has been shown to predict embolic risk associated with intra-cardiac masses such as tumors, thrombi, and vegetations [17, 18]. Sonaglioni *et al*. investigated the use of PW-TDI to assess the prognostic value of LV thrombus mobility in predicting cardioembolic events. Their study indicated that thrombi with mobile free edges and those with peak antegrade velocity equal to or exceeding 10 cm/s were linked to a higher risk of cardioembolic events. This finding suggests that PW-TDI could be instrumental in identifying high-risk patients who might benefit from more aggressive management or closer monitoring [17]. Kal and Garg presented a case involving intracardiac metastasis of a mixed germ cell tumor, diagnosed using multiple imaging modalities, including TEE and Computed Tomography (CT) of the head. This case underscores the importance of a multidisciplinary approach in managing complex cases and highlights the potential role of PW-TDI in evaluating the mobility and behavior of cardiac masses [19]. Although these studies indicate that PW-TDI might be useful for predicting the embolic risk associated with mobile intra-cardiac masses, the current evidence is limited to a few case reports and small-scale studies. Further research is necessary to understand the relationship between PW-TDI and embolic risk fully, and establish standardized protocols for its use in clinical practice. In the present case, incorporating TDI could have enhanced our understanding of the embolic potential of the vegetation, thereby informing more tailored therapeutic strategies.

## CONCLUSION

The diagnosis of Brucellosis can be established based on symptoms and medical examination and confirmed by cardiac imaging and positive serology for Brucella. Although cardiovascular involvement is rare, it has significant outcomes, and patients should be evaluated carefully for cardiac involvement. In conclusion, this report has drawn attention to multivalvular involvement and cardiac rhythm abnormalities in Brucellosis (in our case, A.V. dissociation was present) because early diagnosis and treatment can cause a significant decrease in morbidity as well as mortality by appropriate treatment.

## Data Availability

The data and supportive information are available within the article.

## LIST OF ABBREVIATIONS

CT: Computed Tomography
E.C.G.: Electrocardiography
LV: Left Ventricular
PW: Pulsed Wave
S.A.T.: Serum Agglutination Titre
T.T.E.: Transthoracic Echocardiography
TDI: Tissue Doppler Imaging

## STANDARDS OF REPORTING

CARE guidelines were followed.
